# Acute disseminated actinomycosis presenting as pneumonia with bilateral pulmonary nodules and pelvic osteomyelitis in an immunocompetent patient

**DOI:** 10.1016/j.idcr.2022.e01540

**Published:** 2022-06-18

**Authors:** Sachin M. Patil, Phillip Paul Beck, Maryna Vaznitsel, Andres Bran-Acevedo, Michael Hunter, Jonathan Ross Ang, William Roland

**Affiliations:** a Department of Medicine, Division of Pulmonary, Critical Care and Environmental Medicine, USA; b Department of Medicine, Division of Infectious Diseases, USA; c Department of Pathology, PGY4 Fellow, University of Missouri Health Care, USA

**Keywords:** Disseminated, Actinomycosis, *Actinomyces odontolyticus*, Pneumonia, Pulmonary nodules

## Abstract

Actinomycosis is an indolent human infectious disease caused by gram-positive anaerobic filamentous bacteria *Actinomyces*. Despite its sluggish growth, clinical manifestations can be acute or chronic. Over the last five decades, a significant incidence decline in the western world is due to the discovery of effective antimicrobials and improved oral hygiene. Actinomycosis is now rarely encountered and often misdiagnosed as its manifestations mimic malignancy and other infectious diseases. Due to prior use of antimicrobials, laboratory diagnostic processes often fail to isolate the organism making it arduous to establish the diagnosis. Clinical classification is based on the geographical distribution of the disease as oro-cervicofacial, thoracic, abdominopelvic, neurologic, musculoskeletal, and disseminated. Disseminated and pulmonary actinomycosis in an immunocompetent individual is extremely rare. Here we present a 53-year-old healthy male presenting with acute disseminated actinomycosis with bilateral pulmonary nodules, right upper lobe pneumonia, and pelvic osteomyelitis from *Actinomyces odontolyticus* infection.

## Introduction

*Actinomyces* are gram-positive anaerobic or microaerophilic bacteria causing a clinical disease Actinomycosis. It often causes a slow chronic infection with a rare acute presentation in oro-cervicofacial or soft tissue disease [1]. Although present as mucosal surface commensals in the oropharynx, gastrointestinal tract, and urogenital tract, *Actinomyces* cannot breach the mucosal barrier to cause an infection. Oropharyngeal colonization is complete (100%) by two years of age [2]. The current actinomycosis incidence in the United States of America (USA) is unknown. Prior studies in 1970 mention an incidence of 1:300,000 in the USA and an incidence of 1:100,000 in Holland and Germany [3]. Males are three times more likely to be affected than females due to inadequate oral hygiene and increased risk of oro-facial trauma [3]. Etiologies causing mucosal barrier disruption such as oral trauma, oral surgical procedures, and radiation therapy to the neck and head increase *Actinomyces* infection risks by enabling access to deeper tissues and blood [4], [5]. Recently it has been observed in patients with a lack of healthcare access, bisphosphonates, and intrauterine contraceptive device usage. Certain medications (steroids, anti-TNF- α agents, bisphosphonates), male gender, diabetes mellitus, age (20–60years), alcohol abuse, local tissue damage, and immunosuppression increase the risk for actinomycosis [6], [7]. Improved dental hygiene, medical health care access, and availability of highly effective antimicrobials with an earlier use than before have resulted in a significant decline in the actinomycosis rate. Here we describe a 53-year-old caucasian immunocompetent male with a manifestation of acute disseminated actinomycosis with bilateral pulmonary nodules, right upper lobe pneumonia, and pelvic osteomyelitis due to *Actinomyces odontolyticus* infection.

## Case presentation

**Fig. 1:**
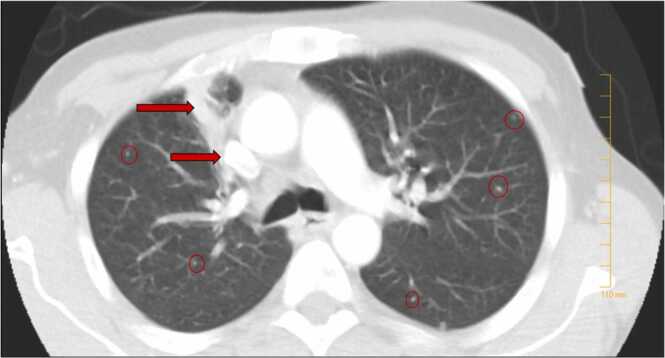
CT of the chest with contrast revealed right upper lobe (RUL) heterogeneous enhancing mass (red arrows) and scattered bilateral nodules <6 millimeters (transparent red rings).

**Fig. 2:**
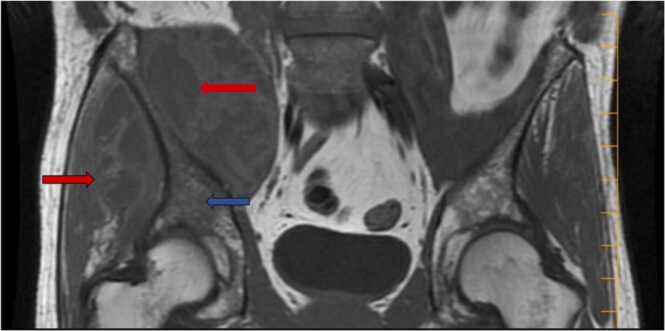
MRI of the pelvis and lumbar spine with and without contrast revealed a large multilobulated cystic mass 17 × 11.8 × 10.5 cm (red arrow) centered around the right iliac bone (blue arrow) with extension medially into portions of the iliopsoas muscle, laterally into the adjacent gluteal musculature, and posterosuperiorly into the posterior paraspinal musculature.

A 53-year-old male with a clinical history of nephrolithiasis came to the outside hospital emergency room (ER) with severe pain in the low back, right hip, and thigh, intermittent fever with chills and night sweats, minimally productive intermittent cough spells with whitish phlegm, exertional dyspnea, generalized fatigue for three weeks along with weight loss of 20 pounds in one month. Six days prior, he was seen at the ER for back pain radiating to the right leg for the last two weeks, and x-rays of the right knee and hip with pelvis were within normal limits. He was discharged home with oral prednisone 40 mg daily for five days and as needed diazepam. He was a farmworker, had quit smoking a month back with 30 pack-years of smoking, and was negative for substance and alcohol abuse. He was sexually active with his girlfriend and resided in a trailer. Clinical examination revealed stable vital signs, lack of sensation over the right anterolateral thigh with no point tenderness, painful hip movements with limited motion, and difficulty walking. Labs revealed leukocytosis, thrombocytosis, hypoalbuminemia, and elevated inflammatory markers (Table 1). He received intravenous (IV) ketorolac 30 milligrams (mg) followed by hydromorphone 0.5 mg 1 dose IV, ondansetron 4 mg IV, and oral diazepam 2 mg. Contrasted computed tomography (CT) of the chest, abdomen, and pelvis revealed right upper lobe (RUL) heterogeneous enhancing mass and scattered bilateral nodules < 6 millimeters (Fig. 1), right iliac diffuse permeative appearance, extensive complex septated acute collections within the right iliopsoas region, deep right gluteal region and the right paraspinal musculature from the L4 - S2 levels. Magnetic resonance imaging (MRI) of the pelvis and lumbar spine with and without contrast revealed a large multilobulated cystic mass centered around the right iliac bone with extension medially into portions of the iliopsoas muscle, laterally into the adjacent gluteal musculature, and posterosuperiorly into the posterior paraspinal musculature (Fig. 2). He was admitted and initiated on pain control, IV fluids, and evaluated by the oncology and orthopedics team, who recommended a biopsy or transfer to a tertiary center. On day two, he was febrile at 39.2^**◦**^Celsius (C) and ordered blood cultures returned negative, whereas procalcitonin and lactic acid were elevated (Table 1). Empirical IV ceftriaxone and azithromycin were started for suspected right upper lobe pneumonia, and his lactic acid improved. Over the next three days, antibiotics were continued, and on day six, he was transferred to our institution for suspected primary bone malignancy with metastasis.

**Table 1:** Outside hospital labs.

1) White cell count (4800–10,800/mL)	22,600
2) Platelet count (130,000–440,000/mL)	679,000
3) Albumin (3.4–5 gm/dL)	2.2
4) Urine analysis with microscopy	Negative for Urinary tract infection
5) Erythrocyte sedimentation rate (0–15 mm/Hr)	90
6) C-Reactive protein (0.0 – 0.30 mg/dL)	16
7) Lactic acid (0.4–20 mmol/L)	4.15 improved to 1.5
8) Procalcitonin (< 0.5 ng/mL)	4.63
9) Lactate dehydrogenase (87–241 U/L)	119
10) Uric acid (3.5–7.2 mg/dL)	4.6

**Fig. 3:**
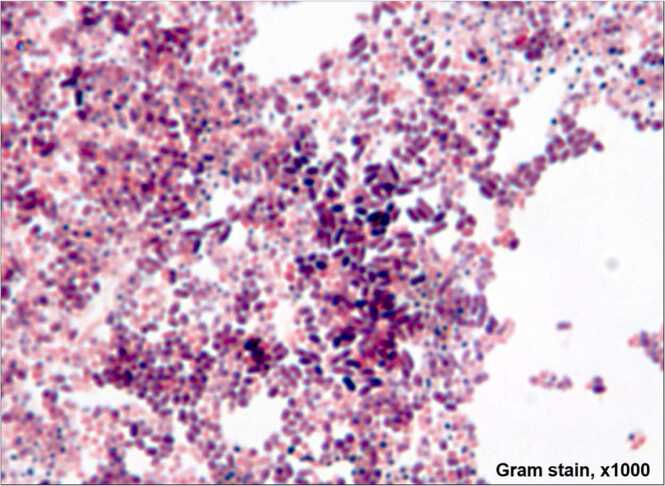
Right pelvic fluid collection revealing bacterial colonies most consistent with Actinomyces.

**Fig. 4:**
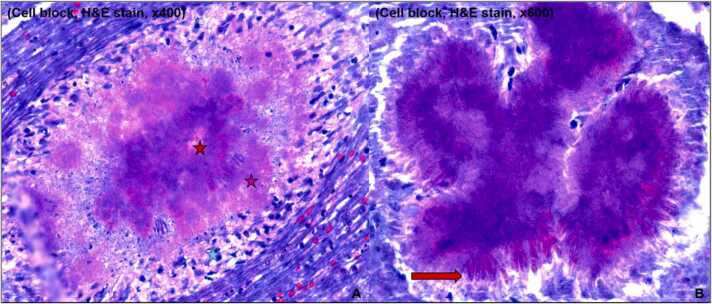
(A). Right pelvic fluid histopath revealed Splendore-Hoeppli phenomenon [Sulfur granules (Clumps of bacterial forms with a basophilic center (red star), eosinophilic periphery (pink star) and associated inflammation (blue star). (B). Sulfur granule revealing swollen terminal process or clubs with proteinaceous debris and eosinophilic material (Antigen-antibody complexes and debris from the inflammatory cells of the host).

On arrival, clinical examination revealed fever (39^◦^C), mild improvement of the right hip movements, and poor oral hygiene. Labs revealed leukocytosis, thrombocytosis, and two sets of blood cultures returned negative (Table 2). IV antibiotics were changed to vancomycin and piperacillin-tazobactam. He then underwent an interventional radiology-guided biopsy of the right ilium and aspiration of 20 mL purulent fluid from the right gluteal mass as recommended by the orthopedic oncology team. He remained intermittently febrile over the next two days, which gradually subsided with his right hip pain improvement. The bone biopsy revealed extensive acute and chronic inflammation with focal granulomatous change and no malignancy. The aspirated purulent fluid showed extensive necroinflammation, bacterial colonies most consistent with *Actinomyces* (Fig. 3) and no malignant cells. Right pelvic fluid histopath revealed the Splendore-Hoeppli phenomenon (sulfur granules and associated inflammation) (Fig. 4). The infectious disease (ID) team recommended continuing the piperacillin-tazobactam and stopping the vancomycin. Inflammatory markers were still elevated, whereas Quantiferon Gold Tuberculosis testing and serology for human immunodeficiency virus were negative (Table 2). A repeat contrasted CT scan of the chest, abdomen, and pelvis confirmed the prior pulmonary findings, whereas, in the pelvis, there was an interval increase in the size of the complex cystic mass indicative of abscess formation. He then underwent irrigation and debridement of the right hemipelvis and an open right ilium biopsy. Right ilium biopsy returned positive for acute and chronic osteomyelitis and negative for cancer. The right pelvic mass biopsy revealed fibrovascular tissue with acute and chronic inflammation, necrosis, hemorrhage, fibrosis, and negative for malignancy. Intraoperative tissue bacterial, mycobacterial and fungal cultures were negative. The pulmonary team performed the bronchoscopy and obtained an RUL protective specimen brush culture sample, bronchioalveolar lavage (BAL) for analysis, culture, and endemic fungal workup. Endobronchial ultrasound (EBUS) guided fine-needle aspiration on enlarged lymph nodes (stations 11 L,7, and 4 R) returned negative for infection and malignancy. BAL fluid analysis did not suggest any infection; however, the cultures returned positive for *A. odontolyticus* (Table 2). *A. odontolyticus* was sensitive to penicillin and resistant to clindamycin. Piperacillin-tazobactam was changed to ceftriaxone 2 g (gm) daily.

**Table 2:** Labs at our institution.

1) White cell count (3500–10,500/mL)	21,300
2) Platelet count (150,000–450,000/mL)	525,000
3) Erythrocyte sedimentation rate (0.0 – 20 mm/Hr)	68
4) C-Reactive protein (0.0–0.5 mg/dL)	10.94
5) Albumin (3.5–5.2 gm/dL)	1.9
6) Human immunodeficiency virus serology	Nonreactive
7) Tuberculosis Gold QuantiFERON test	Negative
8) Outpatient Erythrocyte sedimentation rate (0.0–20 mm/Hr)	34
9) Outpatient C-Reactive protein (0.0–0.5 mg/dL)	0.59
10) Bronchioalveolar fluid analysis (BAL)	
i) Appearance	Clear
ii) Color	Colorless
iii) Neutrophil	23%
iv) Lymphocytes	12%
v) Monocytes/macrophages	62%
vi) Eosinophil	3%
vii) BAL white cell count	130/mcL
viii) BAL red blood cells	<3000/mcL
ix) BAL Histoplasma antigen	Negative
x) BAL Blastomyces antigen	Negative
xi) BAL Aspergillus antigen index	<0.500
xii) BAL Mycobacterial culture	Negative
xiii) BAL Fungal culture	Negative
xiv) BAL bacterial culture	*Actinomyces odontolyticus*

**Fig. 5:**
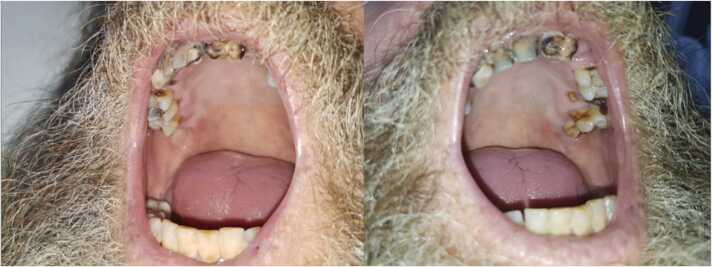
Oral cavity revealing poor oral hygiene with extensive dental caries and tooth decay.

An oral dental surgeon evaluated his bad oral hygiene (Fig. 4). A transthoracic echocardiogram displayed an ejection fraction of 60% with normal cardiac valves and normal left and right ventricular systolic function. An X-ray Panorex revealed several missing teeth, multilevel caries, significant maxillary and mandibular lamina propria resorption, periapical lucency involving the remaining right maxillary molar, and possible retention cyst/opacification of the left maxillary sinus. He was recommended outpatient follow-up in three weeks to fix his bad teeth. A repeat MRI pelvis with contrast revealed a marked interval decrease in size of the complex multiloculated abscess involving the right retroperitoneum, gluteal muscles, and right paraspinal muscles, abnormal marrow signal, and osseous erosion of the right ilium. He was discharged home on IV ceftriaxone 2 gm daily to complete six weeks of antimicrobial therapy. Four weeks later, he followed up at the ID clinic with a decline in inflammatory markers (Table 2), and he was transitioned to oral amoxicillin 875 mg thrice a day for the next 12 months. Repeat CT chest with contrast at eight weeks post-discharge revealed resolving right upper lobe consolidation with residual linear opacities. A repeat MRI pelvis with and without contrast 12 weeks after discharge revealed markedly improved osteitis of the right innominate bone, surrounding soft tissue infection, and minimal persistent marrow changes within the right iliac bone. At the ninth-month ID clinic follow-up, he had his teeth fixed, recovered weight loss, and resumed daily activities. He completed 12 months of oral amoxicillin with complete recovery. (Fig. 5).

## Discussion

Actinomycosis is subclassified into different types based on the site of infection, and these include oro-cervicofacial, thoracic, abdominopelvic, neurologic, musculoskeletal, and disseminated disease. The most common area affected is the oro-cervicofacial (50%), and the least common is the disseminated with multiple organ involvement [7], [8]. Disseminated actinomycosis is an infrequent clinical presentation in the modern antimicrobial era, and almost all subspecies of *Actinomyces* are capable of it. The *Actinomyces* subspecies frequently causing this are *A. meyeri*, *A. israeli*, and *A. odontolyticus* [9]. Often the pathophysiology involves hematogenous dissemination from any infectious site affecting multiple organs simultaneously. *Actinomyces* avoid phagocytosis by the host immune system by coalescing together as one, and concurrent infection with fellow oral commensal help in biofilm formation [10], [11]. Most actinomycotic infections are polymicrobial with simultaneous synergistic involvement of the oral commensal [12]. The commonly affected organs include the lung and the liver, with multiple nodular manifestations simulating a malignancy [13]. Pulmonary actinomycosis occurs due to aspiration, hematogenous, or rarely contiguous spread from the adjacent areas. Clinical manifestations include pneumonia, relapsing pneumonia, cavitatory pneumonitis, pleural thickening, pleural effusion, or empyema [14]. Infrequent clinical presentations include pulmonary nodules, endobronchial lesions, and miliary pattern. Radiological features indicative of possible thoracic actinomycosis include extension across pleura, fissures, mediastinal and adjacent bony disease [15]. Pelvic actinomycosis in the absence of abdominal or genitourinary complaints in a male points to a hematogenous source of dissemination. Often the pelvic illness is due to contiguous spread from the abdominal or genitourinary sites of infection.

CT, MRI, and Fluoro-Deoxy Glucose- Positron Emission Tomography are often used to identify the infection extent and ideal sites to obtain clinical specimens for diagnosis [16]. Diagnostic methods include the demonstration of filamentous anaerobic gram-positive bacilli on a gram stain, sulfur granules (Splendore-Hoeppli phenomenon) on the hematoxylin-eosin stain, macroscopic granules in clinical specimens, and isolating the organism on a culture. New techniques such as polymerase chain reaction-based 16S rRNA (Ribosomal nucleic acid) sequencing are frequently used in clinical labs for identification [17]. A critical point to be considered is the high failure rate in microbial growth (> 50%) due to prior antimicrobial use and poor technique [3]. Treatment is highly individualized based on the site involved and the extent of spread. Often the treatment duration is around six to twelve months, and in most cases, medical therapy alone is adequate. With or without beta-lactamase inhibitors, oral penicillins are the drug of choice [6], [13]. In case of penicillin allergy, erythromycin or tetracyclines can be used. For parenteral use in patients with osteomyelitis or intracranial lesions, a daily higher dose of ceftriaxone is an effective regimen based on anecdotal clinical experience [6]. Clindamycin and metronidazole monotherapy should be avoided due to higher resistance rates (> 20%) [13], [18]. Surgical resection is rarely needed in refractory or extensive disease.

Our patient had no significant clinical comorbidities except poor oral hygiene, increasing his risk of actinomycosis. His initial clinical presentation was similar to a primary bony neoplasm with extensive metastasis. His pelvic and pulmonary disease were possibly due to hematogenous dissemination of the oral disease. His pulmonary presentation was rare due to the presence of bilateral pulmonary nodules along with a right upper lobe mass with heterogeneous enhancement. His pelvic disease was extensive, with no intraabdominal or genitourinary infection. Another interesting observation in our patient was the BAL cultures positivity even after two weeks of therapy which is rare. In the absence of a fistula, his treatment duration was prolonged (12 months after completion of parenteral ceftriaxone) due to the extensive disease. He responded excellently to the intraoperative debridement and antimicrobials with complete recovery.

## Conclusion

Our patient had disseminated actinomycosis involving the pelvic and pulmonary sites. Disseminated actinomycosis is a rare clinical manifestation with the source often from the oral cavity. Clinicians should be aware of actinomycosis mimicking malignancy and consider it in the differential. Actinomycosis is a diagnostic conundrum as it has become infrequent with limited physician experience and difficulties associated with laboratory detection. Gram stain and histopathology findings supplement the microbial isolation in diagnosis. Due to significant resistance, metronidazole and clindamycin should not be used as empirical agents. Even with substantial dissemination, the clinical disease is often curative with effective antimicrobial therapy with earlier detection.

## Consent

Written and verbal consent was obtained from the patient for this report.

## Ethical approval

Not required.

## Funding

This case report has received or utilized no external funding.

## Conflict of interest

The authors declare that they have no conflicts of interest.
